# Analysis of an independent tumor suppressor locus telomeric to *Tp53* suggested *Inpp5k* and *Myo1c* as novel tumor suppressor gene candidates in this region

**DOI:** 10.1186/s12863-015-0238-4

**Published:** 2015-07-14

**Authors:** Carola Hedberg Oldfors, Diego Garcia Dios, Anna Linder, Kittichate Visuttijai, Emma Samuelson, Sandra Karlsson, Staffan Nilsson, Afrouz Behboudi

**Affiliations:** Department of Medical and Clinical Genetics, Sahlgrenska Academy, University of Gothenburg, SE-40530 Gothenburg, Sweden; Tumor Biology Research Group, School of Bioscience, University of Skövde, SE-54128 Skövde, Sweden; Institute of Mathematical Statistics, Chalmers University of Technology, SE-41296 Gothenburg, Sweden

**Keywords:** Endometrial carcinoma, 17p13.3, RNO10q24-q25, *Tp53*, *Hic1*, *Inpp5k*, *Skip*, *Myo1c*

## Abstract

**Background:**

Several reports indicate a commonly deleted chromosomal region independent from, and distal to the *TP53* locus in a variety of human tumors. In a previous study, we reported a similar finding in a rat tumor model for endometrial carcinoma (EC) and through developing a deletion map, narrowed the candidate region to 700 kb, harboring 19 genes. In the present work real-time qPCR analysis, Western blot, semi-quantitative qPCR, sequencing, promoter methylation analysis, and epigenetic gene expression restoration analyses (5-aza-2´-deoxycytidine and/or trichostatin A treatments) were used to analyze the 19 genes located within the candidate region in a panel of experimental tumors compared to control samples.

**Results:**

Real-time qPCR analysis suggested *Hic1* (hypermethylated in cancer 1), *Inpp5k* (inositol polyphosphate-5-phosphatase K; a.k.a. *Skip*, skeletal muscle and kidney enriched inositol phosphatase) and *Myo1c* (myosin 1c) as the best targets for the observed deletions. No mutation in coding sequences of these genes was detected, hence the observed low expression levels suggest a haploinsufficient mode of function for these potential tumor suppressor genes. Both *Inpp5k* and *Myo1c* were down regulated at mRNA and/or protein levels, which could be rescued in gene expression restoration assays. This could not be shown for *Hic1*.

**Conclusion:**

*Innp5k* and *Myo1c* were identified as the best targets for the deletions in the region. *INPP5K* and *MYO1C* are located adjacent to each other within the reported independent region of tumor suppressor activity located at chromosome arm 17p distal to *TP53* in human tumors. There is no earlier report on the potential tumor suppressor activity of INPP5K and MYO1C, however, overlapping roles in phosphoinositide (PI) 3-kinase/Akt signaling, known to be vital for the cell growth and survival, are reported for both. Moreover, there are reports on tumor suppressor activity of other members of the gene families that *INPP5K* and *MYO1C* belong to. Functional significance of these two candidate tumor suppressor genes in cancerogenesis pathways remains to be investigated.

**Electronic supplementary material:**

The online version of this article (doi:10.1186/s12863-015-0238-4) contains supplementary material, which is available to authorized users.

## Background

Allelic loss at 17p is one of the most frequently reported chromosomal alterations in a variety of human malignancies [1–4]. There are quite a few tumor suppressor loci reported in this region among which *TP53* on 17p13.1 is the most prominent one reported to be altered in over 40 % of all tumors [5]. However, several studies clearly provided evidence for presence of an independent, commonly deleted region or regions at 17p13.3, suggesting the existence of an additional tumor suppressor gene(s) distal to the *TP53* locus [6–9]. Despite many efforts, no definite candidate(s) has yet been identified.

We have previously reported a similar observation of an independent tumor suppressor locus distal to *Tp53* in an experimental model for endometrial carcinoma (EC) [10]. Cytogenetic and molecular analysis of ECs developed in a rat model for this malignancy revealed frequent allelic losses/deletions in the proximal to middle part of rat chromosome 10 (RNO10) [11–13]. Through deriving onco-tree models based on allelic imbalance (AI) data, we determined the likely order of allelic loss events along RNO10 as well as their relationship to each other [14]. In the analysis one of the small regions of recurrent allelic loss located at RNO10q24-q25 was placed closest to the root of the onco-tree models, suggesting this region to harbor early and important genetic alterations. The classical tumor suppressor gene *Tp53* is located close to this chromosomal segment and thus was selected as the candidate. Subsequent analysis, however, revealed that *Tp53* was not the only target [10], and in fact, the observed pattern for AI, chromosomal breaks and deletions suggested that major selection was directed against a region located close to, but distal of *Tp53*. This independent, commonly deleted chromosomal segment at RNO10q24-q25 is homologous to the frequently reported loci of tumor suppressor activity on 17p13.3 in several human malignancies [6–9]. Using the experimental tumor model, we developed a detailed deletion map and narrowed down the size of the region to a chromosomal segment of about 700 kb [10]. There are 19 genes located in this segment, including several putative tumor suppressor genes, notably *Hic1* (hypermethyalted in cancer 1), *Ovca1* (ovarian cancer-associated gene 1), and *Ovca2* (ovarian cancer-associated gene 2) [9]. In the present work, we subjected the 19 genes located in this candidate region to expression analysis in a panel of rat EC and non-malignant endometrium samples. Statistical analysis of qPCR results combined with subsequent gene mutation screening along with epigenetic and protein expression analyses suggested *Inpp5k* and *Myo1c* as the most prominent target tumor suppressor candidates in this region.

## Results

### Real-time quantification PCR

**Fig. 1:**
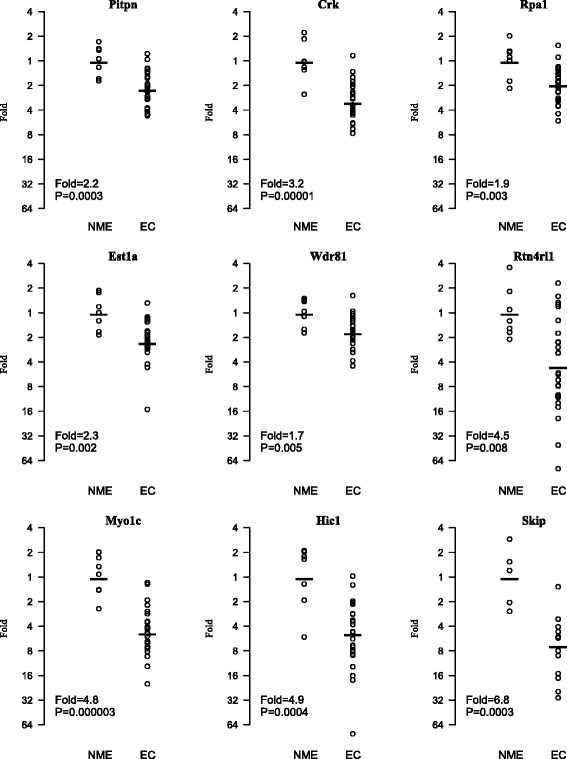
Results of Real-time RT-PCR analysis revealed significant reduced expression of nine genes (nominal *P* value < 0.05) in a panel of 28 rat endometrial carcinomas (EC) compared to seven non-malignant (NME) samples. NME, non-malignant endometrium; EC, rat EC tumors; Fold (fold changes): magnitude of difference in gene expression between the two groups of samples (EC vs NME); *P*, nominal P value

To determine potential target genes for the observed frequent AI/deletions distal to the *Tp53* gene [10], we determined the expression profile of all the 19 genes located in this region in a panel of 28 rat primary tumor and seven NME (non-malignant endometrium) cell cultures. Nine of the genes displayed significant decreased expression in EC compared to the NME samples (nominal *P*-value < 0.05, Fig. 1, Table 1). The question was then whether the observed reduced expression of these nine genes was due to the physical deletion of the genetic material or other regulatory mechanisms. To address this, we used earlier CGH, AI, and FISH data [12, 13, 15, 16] and divided the 28 tumors analyzed in the gene expression assays into two groups: ECs with deletion/AI and those without deletion/AI spanning the 700 kb candidate chromosomal segment. We subsequently used this new grouping of tumors and re-analyzed the real-time RT-PCR results to determine whether there existed a correlation between physical deletion and the observed lowered expression of the nine genes among the tumor groups. Lowered expression of five genes (*Hic1*, *Rpa1*, *Inpp5k*, *Myo1c* and *Crk*) was found to lack correlation with the physical deletion in the region (Table 1), suggesting the involvement of other regulatory mechanisms in silencing of these genes. Fold change in expression of *Rpa1* was minimal and *Crk* is mostly known as an oncogene. The remainder three genes were thus selected as candidates for further analysis.

**Table 1:** Changes in relative expression of 19 genes within tumor suppressor region located at 62.3 – 63.0 Mb (RNO10q24) distal to *Tp53*

Transcript	Position (Mb)	Group EC vs NME			Group EC with vs EC without loss at RNO10q24	
		P-value	<0.05	Fold change	P-value	<0.05
Est1a	62.23	0.002	x	−2.3	0.03	x
Hic1	62.47	0.0004	x	−4.9	**0.2**	
Ovca2	62.48	0.760				
Dph1	62.49	0.973				
Rtn4rl1	62.50	0.008	x	−4.5	0.02	x
Rpa1	62.61	0.003	x	−1.9	**1.1**	
Smyd4	62.66	0.918				
Serpinf1	62.71	0.293				
Serpinf2	62.75	0.324				
Wdr81	62.76	0.00.5	x	−1.7	0.01	x
Tlcd2	62.79	0.218				
Prf8	62.81	0.054				
Rilp	62.83	0.108				
Scarf1	62.84	0.811				
Slc43a2	62.85	0.247				
Pitpn	62.91	0.0003	x	−2.2	0.0004	x
Inpp5k	62.95	0.0003	x	−6.8	**0.2**	
Myo1c	62.99	0.000003	x	−4.8	**0.1**	
Crk	63.02	0.00001	x	−3.2	**0.4**	

Nine genes displayed significant decreased expression (nominal p-value < 0.05) in EC compared to the NME samples. Tumors were then divided into two groups of with and without deletion/AI at RNO10q24 and the gene expression data for the nine down regulated genes were reanalyzed. Down regulation of five genes (marked in bold) was not correlated with physical deletion at RNO10q24

Fold changes and P-values of the different transcripts. The negative values of fold change represent a decreased expression. FC shown only for genes displaying P-values <0.05 between NME vs EC

Equal variances assumed. t-test for equality of means

### Western blot analysis of Hic1, Inpp5k and Myo1c expressions

Based on qPCR results, five rat ECs with differential expressions of Hic1, Inpp5k and Myo1c and from different genetic backgrounds as well as three NME samples, as control (Additional file 1: Table S1), were selected for Western blot analysis to validate the qPCR data. The analysis revealed no decrease in the expression of Hic1 protein in EC compared to the NME samples (Fig. 3a and 3b). The Myo1c protein expression levels correlated well with the observed qPCR fold changes and down regulation of Myo1c protein expression was detected in four out of five ECs compared to the NME samples (Fig. 3a and 3b). Our attempts for expression analysis of Inpp5k protein in this sample set failed, most likely due to the problem with specificity of the purchased antibody for rat samples that is produced against the human INPP5K protein.

### DNA sequencing and mutation analysis of Hic1, Inpp5k and Myo1c

To screen *Hic1, Inpp5k and Myo1c* genes for potential inactivating mutations, the entire coding regions of *Hic1*, *Inpp5k* and *Myo1c*, as well as the promoter region of *Myo1c* were sequenced using genomic DNA from tumor cell cultures and the parental rat strains (BDII, BN and SPRD) as template (Additional file 1: Table S1). No mutation in *Hic1*, *Inpp5k* or *Myo1c* was found.

Sequence analysis of *Hic1* and *Myo1c* revealed 10 strain specific variations within these genes: five in *Hic1* (two in noncoding and three in coding sequences, Additional file 2: Table S5) and five in *Myo1c* (all in noncoding sequences, Additional file 2: Table S5). It is important to note that one of the identified SNPs in the coding sequence of *Hic1* gene (at nt 62474413) results in a non-conservative amino acid substitution in BDII strain, from nonpolar alanine (Ala177) to polar threonine (Thr177, Additional file 2: Table S5).

### Methylation analysis of Hic1, Inpp5k and Myo1c

To investigate the potential involvement of epigenetic inactivation of the three candidate genes in tumors without physical deletion, a panel of tumors with or without deletion in the region, and with differential expressions of the Hic1, Inpp5k and Myo1c, were subjected to DNA methylation analysis. The overall methylation profile of CpG islands in *Hic1*, *Inpp5k* and *Myo1c* promoter regions was characterized using the bisulfite genomic sequencing method in a panel of 18 samples, including 14 ECs (Additional file 1: Table S1), three parental strains (BDII, BN and SPRD) and a positive control sample (methylase treated BDII). This analysis was complemented with the MSP method for parts of *Hic1* promoter region for which bisulfite sequencing was not successful (data not shown). Using the CpG Island Searcher program (http://www.uscnorris.com/cpgislands2/cpg.aspx), the promoter region of the *Hic1* gene was found at position −1352 to −476 (877 bp, including 47 CpG sites). Bisulfite genomic sequencing (for 26 CpGs) combined with MSP analysis (for the remainder 21 CpGs) revealed partial promoter methylation in 9 out of 14 (64 %) of the tumors (Fig. 3c). The *Inpp5k* promoter was located at position −517 to +53 (570 bp, containing 53 CpG sites). The location of the promoter region of the *Myo1c* gene was found at position −2000 to −1111 (890 bp, including 55 CpG sites). No methylation was detected in the *Inpp5k* and *Myo1c* promoters in the tumors analyzed, whereas different degrees of promoter methylation were detected in the *Hic1*. The detected *Hic1* promoter methylation was not correlating with the Hic1 expression at RNA and/or protein levels Fig. 3c.

### Restoration of gene expression after 5-Aza-dC and/or TSA treatments

To investigate whether other epigenetic mechanisms might be involved in the reduction of expression of the three candidate genes we subjected a selected panel of cells to restoration of gene expression treatments. We selected four EC cell lines with reduced Myo1c, Hic1, and/or Inpp5k expression (NUT12 and NUT50 with, and NUT51 and NUT98 without deletion/AI in the candidate region RNO10q24-q25) as well as REF (rat embryonic fibroblast) cells for the analysis. Gene expression analysis at both RNA and protein levels, as expected, revealed no restoration of Hic1, Innp5k and Myo1c expressions after 5-Aza-dC and/or TSA treatments in the control cell REF (Fig. 4). The analysis also showed no or a marginal restoration (only after AZA treatment in NUT51) of gene expression for Hic1 after either or both treatments in the tumor samples tested Fig. 4a, d and e). RT-PCR analysis revealed a strong restoration of Inpp5k expression following 5-Aza-dC and/or TSA treatments in three of the tumors analyzed (NUT50, NUT51 and NUT98, Fig. 4b). For the *Myo1c* gene, a marginal restoration of gene expression after the treatments was detected in RT-PCR analysis, in particular for NUT50 (Fig. 4c). Analysis of Myo1c protein, however, was more conclusive and clearly showed that expression of Myo1c protein was partially restored after the treatments, especially in NUT12 (with physical deletion/AI in the region) and NUT98 (without physical deletion/AI in the region) as well as in NUT50 (with deletion/AI in the region) after the combined treatments (Fig. 4e).

## Discussion

In the present work and by using a set of well-characterized experimental EC tumor samples, and through a candidate gene approach, we characterized the frequently reported independent region of tumor suppressor activity in human tumors located telomeric to *TP53*. We subjected all 19 genes located in this chromosomal segment to gene expression analysis in a panel of 28 rat EC and seven control non-malignant endometrium (NME) samples. Nine genes showed significant reduced expression in EC compared to NME samples (Fig. 1, Table 1). Interestingly, two of the known tumor suppressor genes located in this region, *Ovca1* (ovarian cancer-associated gene 1) and *Ovca2*, were not among these.

**Fig. 2:**
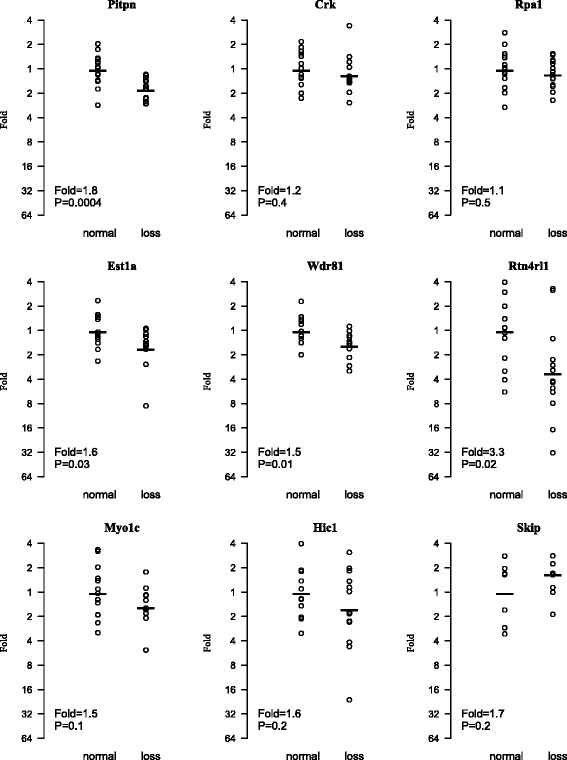
Comparative analysis of relative expression of nine genes between groups of EC tumors with and without chromosomal deletion (14 tumors in each group) in the candidate region. Five genes (*Hic1*, *Rpa1*, *Inpp5k*, *Myo1c* and *Crk*) were almost equally down regulated in both groups of tumors (nominal *P* value > 0.05), suggesting reduction in expression of these genes was not correlated with physical deletion at the candidate chromosomal region and thus regulated by other mechanisms

The question was then whether the observed reduced expression of these nine genes was due to the physical deletion of the genetic material or other regulatory mechanisms. To address this, we used earlier CGH, AI, and FISH data [12, 13, 15, 16] and divided the 28 tumors analyzed in the gene expression assays into two groups: ECs with deletion/AI and those without deletion/AI spanning the 700 kb candidate chromosomal segment. We subsequently used this new grouping of tumors and re-analyzed the real-time RT-PCR results to determine whether there existed a correlation between physical deletion and the observed lowered expression of the nine genes between the tumor groups (Fig. 2, Table 1). Our interest was to identify gene(s) showing no such correlation, indicating that the observed reduction in expression of these gene(s) was not only due to the physical deletion of the genetic material. The results revealed that lowered expression levels of five genes, *Hic1*, *Rpa1*, *Inpp5k*, *Myo1c* and *Crk* were independent of the observed physical deletions (Fig. 2, Table 1). Among these, *Hic1*, *Inpp5k* and *Myo1c* showed strong and highly significant down regulation in EC compared to the control samples (Table 1) and thus were selected as the best candidates. *Rpa1* and *Crk* were not appealing as candidates: *Rpa1* showed only a rather moderate down regulation in gene expression analysis (Table 1) and *Crk* (v-crk sarcoma virus CT10 oncogene homolog, avian) is mainly recognized as an oncogene in cancer studies [17–19].

In qPCR experiments, we found that *Hic1*, *Innp5k* and *Myo1c* were always expressed in the tumors, although at very low levels. According to Knudson’s theory of inactivation of tumor suppressor genes [20], if *Hic1*, *Inpp5k* and *Myo1c* are to behave as classical tumor suppressor genes, it is expected that both alleles of the genes be inactivated in the tumor material. We, therefore, hypothesized that the remaining allele of these genes might have become inactivated through mutation/s. However, in gene sequencing experiments, no mutation was found in coding sequences of these three genes, suggesting a potential haploinsufficient mode of function for these candidate tumor suppressor genes. In gene sequencings we identified ten SNPs in intronic, noncoding and/or coding sequences of the *Hic1* and *Myo1c* genes (Additional file 2: Table S5). Identification of these SNPs offered the possibility to confirm our earlier AI/deletion data [11, 13] and also to make a detailed deletion map with information on the homozygote/hemizygote pattern of the observed deletions spanning the candidate region in the tumors.

**Fig. 3:**
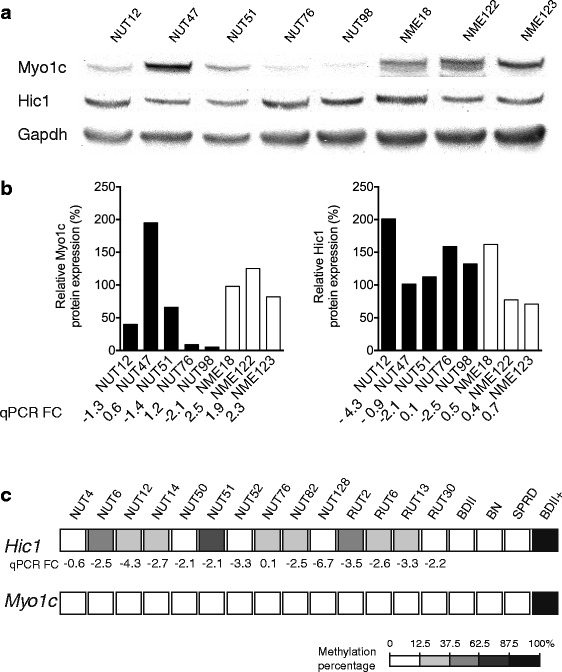
**a** and **b** Analysis of Myo1c and Hic1 protein expression in five EC and three NME samples. The results shown are representative of three independent experiments. Myo1c protein displayed down regulated in four out of five ECs tested, whereas Hic1 protein was not down regulated in the EC samples, even in those that in qPCR experiment showed a strong lowered expression of Myo1c transcript. The analysis additionally showed a very good correlation between expression of Myo1c at RNA and protein levels in the tumors tested, whereas such correlation was not detected for Hic1. EC: enodemetrail carcinoma; NME: non-malignant endometrium. **c** Methylation status of CpG islands in promoter regions of *Hic1* (26 CpG sites) and *Myo1c* in a panel of 14 ECs, three parental strains (BDII, BN and SPRD) and one positive control (BDII +) samples. Methylation frequencies are color coded (black, ranges of gray and white) in five white/gray/black grades corresponding to percentages of CpG sites that were found methylated in each sample. For comparison, qPCR results for the *Hic1* gene is presented as fold changes (FC) for each tumor, where negative and positive fold change values represent reduced and increased expressions, respectively. As shown, a correlation between methylation score at the *Hic1* promoter and expression of this gene in tumors is lacking. No methylation was identified in the promoter region of *Myo1c*

Our interest was then to determine whether gene products of these three candidate genes were down regulated in the tumors. To this end, we examined expression of Hic1, Innp5k and Myo1c proteins in a selected panel of five ECs compared to three NME samples using Western blot. The result for Myo1c showed a good correlation between the qPCR results and protein expression level in the tumor samples, indicating that Myo1c protein was in fact down regulated in the majority of EC tumors tested compared to the control samples (Fig. 3a and b). However, such a correlation was not detected for Hic1 as it was shown that expression of Hic1 protein was not down regulated in the tumors, even in those showing a rather strong Hic1 down regulation at their mRNA level (NUT12, NUT51 and NUT98; Fig. 3a and b). No result was obtained for the Inpp5k protein, most likely due to the lack of specificity of the available human antibody for the rat samples.

There were a number of tumors that did not harbor AI/deletion in the candidate region, but nevertheless displayed a significant down-regulation of Hic1, Inpp5k and/or Myo1c (Table 1). The question was then whether other regulatory machineries, namely epigenetic regulation, were involved. To address this, we screened promoter regions of *Hic1*, *Inpp5k* and *Myo1c* for methylation at CpG islands. No methylation was detected in the *Inpp5k* and *Myo1c* promoter regions in any of the tumors tested, whereas partial DNA methylation was detected in *Hic1* promoter in 64 % of tumors tested (Fig. 3c). No correlation, however, between the observed methylation status of *Hic1* promoter and the level of Hic1 transcript was detected in tumor samples (Fig. 3c).

**Fig. 4:**
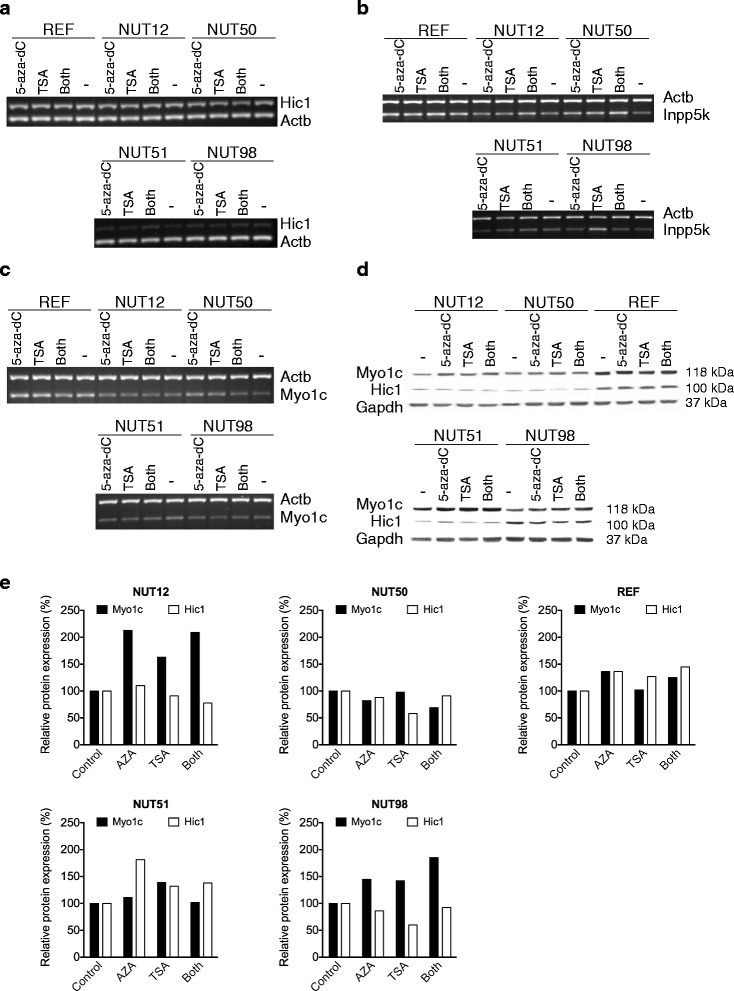
Expression patterns of Hic1, Inpp5k and Myo1c after treatments with 5-Aza-dC and/or TSA in four tumors (NUT12 & NUT50 with and NUT51 & NUT98 without deletion in the candidate region) and one control (rat embryonic fibroblast, REF) samples. The results shown are representative of two independent experiments. **a** Hic1 mRNA expression, **b** Inpp5k mRNA expression, **c** Myo1c mRNA expression, **d** protein expression of Hic1 and Myo1c, and **e** quantification of Western blot analysis for Hic1 and Myo1c expressions. As shown, expression of Hic1 was not specifically restored in these assays (only after AZA treatment in one sample, NUT51), whereas expressions of Myo1c (at both mRNA and protein levels) and Inpp5k (at mRNA level) were restored after either or both of these treatments. Interestingly, restoration of Myo1c gene expression was observed in both tumors with (NUT12) and without (NUT98) AI/deletion in the region

It is quite common that genes with critical functions are under restricting regulation by several promoters. Moreover, histone deacethyaltion is an alternative epigenetic regulation mechanism that may also result in the gene silencing. To examine whether other potential promoter(s) might have been involved in the expression regulation of *Hic1*, *Inpp5k* and *Myo1c* as well as to study the potential involvement of histone deacethylation in expression regulation of these genes, we treated cell lines with demethylating agent 5-Aza-dC and/or deacetylating inhibitor TSA. Surprisingly, Hic1 expression was not specifically restored after the treatments in the samples, especially not in those that showed partial methylation in the Hic1 promoter region (NUT12 and NUT51, Figs. 3c, 4a, d and e). This result suggested that the observed methylation pattern in the Hic1 promoter region in EC tumors, rather than being an epigenetic silencing mechanism, is likely to be a general structural feature for this gene. In agreement with this, dense hypermethylation of one of the *HIC1* alleles has earlier been reported in a number of normal human tissues, including kidney [21], and histologically normal and benign hyperplastic prostate tissues [22].

Restoration of gene expression was detected for Myo1c and Inpp5k genes in the tumor samples at RNA and/or protein levels (Fig. 4b-e). Notably, the observed gene expression restoration for Myo1c protein was irrespective of AI/deletion in the candidate region, as NUT12 with AI/deletion and NUT98 without AI/deletion showed strong restoration of Myo1c expression after either or both treatments (Fig. 4e). These data suggest that epigenetic gene silencing might have an important role in inactivation of *Myo1c* and *Inpp5k* tumor suppressor candidates.

To summarize, qPCR analysis of 19 genes located within the commonly deleted region distal to *Tp53* in experimental ECs, suggested *Hic1*, *Inpp5k* and *Myo1c* as the best candidate tumor suppressor genes in this region. No mutation was detected in the coding sequences of the retained alleles, suggesting a potential haploinsufficient mode of function for these candidate tumor suppressor genes. *Hic1*, *Inpp5k*, and *Myo1c* showed reduced expression in tumor samples irrespective of the presence or absence of physical deletion in the candidate region, suggesting the potential involvement of alternative gene silencing regulatory mechanism(s). We found a rather normal expression of Hic1 protein in the tumors, even in those that showed down regulation of Hic1 at the mRNA levels, indicating lack of correlation between expression of Hic1 at mRNA and protein levels. Promoter methylation analysis revealed partial methylation of *Hic1* promoter in a number of tumors. Nevertheless, there was again no correlation between the presence or absence of *Hic1* promoter methylations and gene expression levels in the tumors. Moreover, Hic1 expression could not specifically be restored after treatments with demethylating and histone deacetylase inhibitor agents. In contrast, while promoter methylation was not detected in *Inpp5k* and *Myo1c* promoters, down regulation of Inpp5k and Myo1c were detected in EC tumors, and this could be rescued after the 5-Aza-dC and TSA treatments, even in tumors without AI/deletion in the candidate tumor suppressor region. Taken together, results from the present work exclude *Hic1* as a fitting candidate and provide evidence for *Inpp5k* and *Myo1c* as two attractive candidates for the observed independent tumor suppressor activity at the neighborhood of *Tp53*.

*INPP5K* (inositol polyphosphate-5-phosphatase K, also known as *SKIP*, skeletal muscle and kidney enriced inositol phosphatase) is a member of the inositol polyphosphate 5-phosphatases family [23] with a poorly characterized function *in vivo*. The inositol polyphosphate 5-phosphatases family is known as negative regulators of PI 3-kinase signaling [24]. Analysis of the role of INPP5K in insulin-stimulated cells indicated that endogenous INPP5K might be one of the key regulators of insulin signaling in skeletal muscle and adipocytes for glucose homeostasis [25]. INPP5K was identified as a 5'-inositol phosphatase that hydrolyzes phosphatidylinositol 3,4,5-triphosphate (PI-3,4,5-P_3_) and phosphatidylinositol 4,5-bisphosphate (PI-4,5-P_2_) to negatively regulate intracellular phosphatidylinositol 3-kinase signaling. It is thus suggested that INPP5K exerts its functions through direct binding to PIP_3_ or forming a complex with molecules located downstream of PI 3-kinase. Activated PI 3-kinase generates PIP_3_ that in turn activates the downstream target AKT, which then positively regulates a range of cellular functions, including actin rearrangement, protein synthesis, cell metabolism, cell cycle (G1-S transition) and cell survival [24]. So far, there is no report on potential involvement of *INPP5K* in cancer progression. However, another member of the phosphoinositide phosphatase family, *PTEN*, has already been identified as a haploinsufficient tumor suppressor gene [26] and its inactivation has been implicated in a variety of human cancers, including endometrial carcinoma. Therefore, it is tempting to speculate that *INPP5K* might likewise be potentially involved in carcinogenesis.

The gene adjacent to *Inpp5k,* and the second candidate tumor suppressor gene identified in the present work, is the molecular motor myosin 1c (*Myo1c*). *Myo1c* exerts overlapping functions in phosphoinositide (PI) 3-kinase/AKT (PI3K/AKT) signaling as those of *Inpp5k.* Myosin 1C is a widely expressed vertebrate unconventional myosin-I isoform that concentrates in perinuclear regions, on ruffling cell membranes, and within stereocilia of hair cells. Increasing evidence points to the role of myosin 1c in many signaling cascades, from the integrin-dependent signaling involved in cell migration to the signaling events underlying insulin resistance (reviewed in [27]). Myo1c is a lipid raft-associated motor protein that is specifically involved in the recycling of lipid raft membrane and proteins that regulate plasma membrane plasticity, cell motility and pathogen entry [28, 29]. MYO1C binds tightly and specifically to PIP_2_ [30], an important second messenger involved in a variety of crucial cellular functions, including regulation of the actin cytoskeleton and signal transduction in insulin and AKT signaling pathways. This protein is additionally involved in glucose uptake in muscle and adipocytes through controlling of movement of intracellular GLUT4–containing vesicles to the plasma membrane [31]. It has been shown that insulin-dependent phosphorylation of Myo1c is required for GLUT4 translocation and transport of glucose through phosphoinositide (PI) 3-kinase/AKT pathway [32, 33]. There is no earlier report on potential tumor suppressor activity of this gene, but several other members of the myosin-I gene family have been reported as cancer-related genes, including tumor suppressor gene *MYO18B* in lung, ovarian and colorectal cancer [34] and involvement of *MYO1F* in chromosomal translocation and gene fusion in infant acute monocytic leukemia [35].

## Conclusions

In conclusion, our data suggested *Inpp5k* and *Myo1c* as potential candidates located adjacent to each other within the reported independent tumor suppressor loci distal to *Tp53*. There is no earlier report on their potential involvement in carcinogenesis, but earlier studies have clearly suggested a regulatory role for these genes in PI3K/Akt pathway, which is known to be vital to the growth and survival of cancer cells [36]. Moreover, other members of gene families that *INPP5K* and *MYO1C* belong to are suggested to function as tumor suppressor genes in a variety of cancer types [26, 34]. Details of potential functional contributions of these two genes to cancerogenesis remain to be further investigated.

## Methods

### Animal crosses and experimental tumor material

All animal experiments were approved by the local ethical committee (Institute of Laboratory Animal Science and Central Animal Facility, Hannover Medical School). Animals of the inbred BDII rat strain are genetically predisposed to spontaneous EC, with an incidence of more than 90 % in virgin females before the age of 24 months [37, 38]. EC tumors developed in F1, F2 and backcross (N1) progeny of crosses between BDII females and males from two EC non-susceptible strains, BN/Han and SPRD-Cu3/Han [39, 40], were included in the present study (Additional file 1: Table S1). In some cases no malignant cells were detected in the removed cell mass from animals when pathologically characterized. Chromosomal analysis of these samples revealed only minor numerical chromosomal changes (unpublished data). In this study these tissues represent normal or pre-malignant endometrium and thus are referred to as non-malignant endometrium (NME, Additional file 1: Table S1). At necropsy, tumor specimens were collected from animals for DNA extraction using a standard phenol-based method in the Genepure™ 341 Nucleic Acid Purification System (PE Applied Biosystems) [12]. Small pieces of fresh tumor as well as NME tissues were used to set up primary cell cultures. From the cell cultures, DNA and total RNA were extracted using the GenElute kit (Mammalian Total RNA Kit, Sigma).

### Real-time quantitative PCR

Expression of 19 genes (Table 2) was analyzed in a panel of 28 EC and seven NME samples using Real-time quantitative PCR as described previously [41]. Quantification and normalization of the results was performed by the standard-curve method. Briefly, a standard curve was prepared in each PCR assay for all genes using serial dilutions (1:1, 1:3, 1:9, 1:18, 1:36, and 1:72) of one of the tumor samples (RUT30) and/or a commercially available rat RNA mix (Stratagene, La Jolla, CA, USA). The mean C_T_-value for triplicates was calculated, C_T_ values of the serial dilutions were used to interpolate standard curves for each gene and data therein was used to determine concentration or copy number of each gene in every test sample. To normalize the results, three housekeeping genes (*Gapdh*, β*-actin* and *Rps9*) were included in the analysis, among which *Rps9* (ribosomal protein S9) showed the lowest variations in ∆C_T_ levels regardless of the cell type [41] and thus was selected and used as the internal reference for normalization of expression of all 19 genes in the samples. Logarithmic expression levels were then compared with Welch's t-test for adjusted expression levels based on standard curves as well as expression of *Rps9* in EC tumors compared to NME samples.

**Table 2:** Genes located within tumor suppressor region between 62.3-63.0 Mb on RNO10 at band 10q24 which is homologous to human chromosome band 17p13.3

Start (Mb)	Gene	Description	Accession no.	Assay ID
62.23	*Est1a*	Telomerase-binding protein EST1A (Ever shorter telomeres 1A)	NM_001105808	Rn01466713_m1
62.47	*Hic1*	Hypermethylated in cancer 1	NM_001107021	CM-Hic1
62.48	*Ovca2*	Candidate tumor suppressor OVCA2	NM_001109036	CM-Ovca2
62.49	*Ovca1*	Candidate tumor suppressor OVCA1, Dph2l1	NM_001105809	CM-Ovca1
62.50	*Rtn4rl1*	Reticulon 4 receptor-like 1	NM_181377	Rn01648154_m1
62.61	*Rpa1*	Replication protein A1	NM_001047843	Rn01460703_g1
62.66	*Smyd4*	SET and MYND domain containing 4	NM_001105810	CM-Smyd4
62.71	*Serpinf1*	Serine (or cysteine) peptidase inhibitor, clade F, member 1	NM_177927	Rn00709999_m1
62.75	*Serpinf2*	Serine (or cysteine) peptidase inhibitor, clade F, member 2	NM_001011892	Rn01464596_m1
62.76	*Wdr81*	Wdr81, WD repeat domain 81	NM_001134360	Rn01460094_m1
62.79	*Tlcd2*	TLC domain containing 2	XM_573142.2	Rn01465130_m1
62.81	*Prpf8*	Pre-mRNA processing factor 8	XM_001080695	Rn01472027_g1
62.83	*Rilp*	Rab interacting lysosomal protein	NM_001105811	Rn01465376_g1
62.84	*Scarf1*	Scavenger receptor class F, member 1	NM_001107022	CM-Scarf1
62.85	*Slc43a2*	Solute carrier family 43, member 2	NM_001105812	CM-Slc43a2
62.91	*Pitpna*	Phosphatidylinositol transfer protein, alpha	NM_017231	Rn01464411_m1
62.95	*Skip*	Skeletal muscle and kidney enriched inositol phosphatase	NM_001013859	Rn01466844_g1
62.99	*Myo1c*	Myosin IC	NM_023092	Rn00576538_m1
63.02	*Crk*	V-crk sarcoma virus CT10 oncogene homolog (avian)	NM_019302	Rn00467066_m1
1q12	*Rps9*	Ribosomal protein S9	NM_031108.2	Rn01530912_g1
4q42	*Gapdh*	Glyceraldehyde-3-phosphate dehydrogenase	NM_017008.3	Rn99999916_s1
12p11	*β-actin*	Beta-actin	NM_031144	Rn00667869_m1

TaqMan assays used in quantitative real-time PCR for the genes in the region as well as three housekeeping genes are presented

CM: Custom made

### Western blot

A panel of five EC and three NME samples was analyzed by western blot to examine levels of Hic1, Inpp5k and Myo1c protein expression using 1:500 rabbit anti-Hic1 (H8539, Sigma Aldrich), 1:1000 rabbit anti-Inpp5k (S8948, Sigma Aldrich), 1:600 rabbit anti-Myo1c (HPA001768, Sigma Aldrich), and 1:500 dilution anti-Gapdh (sc-825778, Santa Cruz, Biotechnology) according to the standard protocol (Additional file 1: Table S1, Fig. 3a and b).

### DNA sequencing of Hic1, Inpp5k and Myo1c

A panel of 32 EC tumors was selected for mutation sequencing of *Hic1* and *Myo1c* (Additional file 1: Table S1). The *Inpp5k* gene was sequenced in 18 tumors. Primer pairs were designed using the Primer3 program and synthesized by a commercial supplier (SIGMA-Genosystem, Cambridge, UK). PCR primers set corresponding to the coding sequences of *Hic1*, *Inpp5k* and *Myo1c* genes were amplified and screened for mutations (Additional file 3: Table S2). For the *Myo1c* gene the promoter region was also sequenced.

PCR amplification products were purified using GFX™ PCR DNA and gel Band Purification Kit (Amersham Pharmcia Biotech, Piscataway, NJ). Using ABI PRISM® BigDye® Terminator v1.1 or 3.1 Cycle Sequencing Kit (Applied Biosystems), the purified DNA fragment were subjected to sequencing according to the protocol provided by the manufacturer. Sequencing products were separated on a denaturing polyacrylamide gel on a 3130xl Genetic Analyzer (Applied Biosystems) and analyzed using the software’s Sequencing Analysis v5.2 and SeqScape v2.5 (Applied Biosystems).

### DNA methylation analysis

A panel of 14 tumors was selected for promoter methylation analysis of *Hic1*, *Inpp5k* and *Myo1c* (Additional file 1: Table S1). One μg DNA from 14 tumors as well as the three controls (parental strains: BDII, BN and SPRD) were denatured, treated by sodium bisulfite and purified using Epitect Bisulfite Kit according to manufacturer protocol (Qiagen). A positive control sample was produced through methylase treatment of DNA from BDII. This sample was then sodium bisulfite treated and processed at similar conditions as the test samples. We used web server CpG Island Searcher (URL: http://www.uscnorris.com/cpgislands2/cpg.aspx) to predict location of CpG islands for both genes. Primers suitable for bisulphite sequencing were designed using the Meth-Primer software (URL: http://urogene.org/methprimer/) and BiSearch web server (URL: http://bisearch.enzim.hu/) and synthesized by a commercial supplier (SIGMA-Genosystem, Cambridge, UK). Promoter regions were PCR amplified using two different overlapping sets of primer for *Hic1* and three sets of primers for *Myo1c* using treated DNA as template (Additional file 4: Table S3). In some cases it was necessary to perform semi-nested PCR to produce enough PCR products for sequencings. The methylation status was then analyzed using bisulphite sequencing as described earlier [42].

Our attempts to PCR amplify and sequence the middle part of the *Hic1* promoter (−1082 to −781) failed. This was mainly due to high density of CpGs in the sequence. We thus analyzed methylation status of this part of the *Hic1* promoter using the MSP (methylation specific PCR) technique. Primer pair sequences and PCR conditions are available upon request

### 5-aza-2´-deoxycytidine (5-Aza-dC) and/or trichostatin A (TSA) treatment

Four EC cell lines (NUT12 and NUT50 with and NUT51 and NUT98 without deletion/AI at RNO10q24) with low expression of *Hic1*, *Inpp5k* and/or *Myo1c*, and rat embryo fibroblasts (REF), were selected for the analysis. All cell cultures were treated with the demethylating agent 5-aza-2´-deoxycytidine (5-Aza-dC, Sigma-Aldrich Co, St Louis, MO, USA) and/or with the histone deacetylase inhibitor trichostatin A (TSA; Sigma-Aldrich). Concentrations of the agents for treatments were chosen based on an earlier study (Karlsson et al., unpublished data). Each cell line was seeded at low density in four separate flasks, treated with 5-Aza-dC, TSA, both 5-Aza-dC and TSA, or no treatment. For 5-Aza-dC treatments, cells were grown in medium containing 2.5 μM 5-Aza-dC for 96 hours, with medium and drug being refreshed twice after 48 h and 72 h. For simultaneous 5-Aza-dC and TSA treatments, the procedure was as 5-Aza-dC treatment, with the addition of 300 nM TSA to the culture during the last 16 hours. For the TSA treatment as well as the negative control, medium was refreshed after 48 h and 72 h, with the exception that for the TSA treatment, 300 nM TSA was added to the culture during the last 16 hours. Two independent experiments for all treatments and tumor samples were performed. After 96 hours, total RNA was isolated from all samples using RNeasy Protect Mini kit (Qiagen) according to the manufacturer’s protocol. Protein extraction was performed according to standard protocols.

### Semi quantitative RT-PCR

Reverse–transcription polymerase chain reaction (RT-PCR) was performed on 1 μg of total RNA using QuantiTect® Reverse Transcription Kit (Qiagen) according to manufacturer’s protocol. Semi quantitative RT-PCR was performed for *Hic1*, *Inpp5k* and *Myo1c* using β-actin as internal control in multiplex PCR. Primer pairs were designed using the Primer3 program and were synthesized by a commercial supplier (SIGMA-Genosystem, Cambridge, UK) (Additional file 5: Table S4). PCR amplification was performed for 26 cycles.

## Additional files

Additional file 1: Table S1. Panel of cell cultures used in the present study. Different sets of samples were used for different analysis as: Mut, mutation sequencing (32 EC); qPCR (28 EC and seven NME), Real-time PCR; WB, Western blot (five EC and three NME); PM, promoter methylation (14 EC); AT, 5-Aza-dC/TSA treatments (four EC and one NME). Additional file 2: Table S5. SNPs identified in the gene sequencing analysis based on the build RGSC3.4 data. Additional file 3: Table S2. Primers used for sequencing the genes *Hic1*, *Myo1c* and *Inpp5k*. Additional file 4: Table S3. Primers used for bisulphite sequencing of the promotors of *Hic1* and *Myo1C*. For *Hic1* the predicted CpG island was 877 bp and includes 47 CpG sites. For *Myo1c* the predicted CpG island of 890 bp and includes 55 CpG sites. Additional file 5: Table S4. Primers used for semi quantitative multiplex RT-PCR for Hic1, Inpp5k and Myo1c amplified in a multiplex PCR with β-actin as the internal control.

## Abbreviations

Crk: v-crk sarcoma virus CT10 oncogene homolog, avian
EC: endometrial carcinoma
Hic1: hypermethylated in cancer 1)
Inpp5k: inositol polyphosphate-5-phosphatase K (also known as Skip)
Ovca1: ovarian cancer-associated gene 1
Ovca2: ovarian cancer-associated gene 2
Myo1c: myosin 1c
NME: non-malignant endometrium
Rpa1: Replication protein A1
Skip: skeletal muscle and kidney enriched inositol phosphatase
