# Supplementary material for: Analysis of an independent tumor suppressor locus telomeric to Tp53 suggested Inpp5k and Myo1c as novel tumor suppressor gene candidates in this region
Source: BMC Genet. 2015 Jul 14;16:80. doi: 10.1186/s12863-015-0238-4 (PMC4501283; doi:10.1186/s12863-015-0238-4)
Supplement: Additional file 1: Table S1. — Panel of cell cultures used in the present study. Different sets of samples were used for different analysis as: Mut, mutation sequencing (32 EC); qPCR (28 EC and seven NME), Real-time PCR; WB, Western blot (five EC and three NME); PM, promoter methylation (14 EC); AT, 5-Aza-dC/TSA treatments (four EC and one NME). [file 12863_2015_238_MOESM1_ESM.docx]

**Panel of cell cultures used in the present study.** Different sets of samples were used for different analysis as: Mut, mutation sequencing (32 EC); qPCR (28 EC and seven NME), Real-time PCR; WB, Western blot (five EC and three NME); PM, promoter methylation (14 EC); AT, 5-Aza-dC/TSA treatments (four EC and one NME).

| **Cross** | **Generation^a^** | **Sample designation** | **Sample type^b^** | **Chr. number^c^** | **Assays** |
| --- | --- | --- | --- | --- | --- |
| BDIIxBN | F1 | RUT7 | EC | 43 | Mut |
|  | F2 | RUT25 | EC | 48 | Mut |
|  | N1 | NUT16 | EC | 42 | Mut |
|  | F1 | RUT12 | EC | 51 | Mut, qPCR |
|  | N1 | NUT31 | EC | 67 | Mut, qPCR |
|  | N1 | NUT46 | EC | 56 | Mut, qPCR |
|  | N1 | NUT81 | EC | 42 | Mut, qPCR |
|  | N1 | NUT97 | EC | 52 | Mut, qPCR |
|  | N1 | NUT99 | EC | 67 | Mut, qPCR |
|  | N1 | NUT100 | EC | 67 | Mut, qPCR |
|  | N1 | NUT127 | EC | 70 | Mut, qPCR |
|  | F2 | RUT30 | EC | 61 | Mut, qPCR, PM |
|  | N1 | NUT6 | EC | 60 | Mut, qPCR, PM |
|  | N1 | NUT52 | EC | 42 | Mut, qPCR, PM |
|  | N1 | NUT82 | EC | 78 | Mut, qPCR, PM |
|  | N1 | NUT128 | EC | 64 | Mut, qPCR, PM |
|  | N1 | NUT50 | EC | 64 | Mut, qPCR, PM, AT |
|  | N1 | NUT76 | EC | 43 | Mut, qPCR, PM, WB |
|  | N1 | NUT51 | EC | 71 | Mut, qPCR, PM, WB, AT |
|  | N1 | NUT98 | EC | 52 | Mut, qPCR, WB, AT |
|  | N1 | NME118 | NME | nd | qPCR |
|  | N1 | NME129 | NME | nd | qPCR |
|  | N1 | NME123 | NME | nd | qPCR, WB |
|  | N1 | NME122 | NME | nd | qPCR, WB, AT |
| BDIIxSPRD | N1 | NUT55 | EC | 44 | Mut |
|  | N1 | NUT7 | EC | 46 | Mut, qPCR |
|  | N1 | NUT39 | EC | 39 | Mut, qPCR |
|  | N1 | NUT42 | EC | 44 | Mut, qPCR |
|  | N1 | NUT84 | EC | 41 | Mut, qPCR |
|  | F1 | RUT2 | EC | 38 | Mut, qPCR, PM |
|  | F2 | RUT6 | EC | 58 | Mut, qPCR, PM |
|  | F2 | RUT13 | EC | 62 | Mut, qPCR, PM |
|  | N1 | NUT4 | EC | 62 | Mut, qPCR, PM |
|  | N1 | NUT14 | EC | 44 | Mut, qPCR, PM |
|  | N1 | NUT12 | EC | 59 | Mut, qPCR, PM, WB, AT |
|  | N1 | NUT47 | EC | 48 | Mut, qPCR, WB |
|  | N1 | NME58 | NME | nd | qPCR |
|  | N1 | NME89 | NME | nd | qPCR |
|  | N1 | NME18 | NME | nd | qPCR, WB |

^a^ F1 = strain intercross first generation animals; F2 = intercross second generation animal; N1 = F1 animal back-crossed to BDII strain animal

^b^ EC = endometrial carcinoma; NME = non-malignant endometrium

^c^ Range of common chromosome numbers in tumor.
