# Supplementary material for: Analysis of an independent tumor suppressor locus telomeric to Tp53 suggested Inpp5k and Myo1c as novel tumor suppressor gene candidates in this region
Source: BMC Genet. 2015 Jul 14;16:80. doi: 10.1186/s12863-015-0238-4 (PMC4501283; doi:10.1186/s12863-015-0238-4)
Supplement: Additional file 2: Table S5. — SNPs identified in the gene sequencing analysis based on the build RGSC3.4 data. [file 12863_2015_238_MOESM2_ESM.docx]

**Primers used for sequencing the genes *Hic1*, *Myo1c* and *Inpp5k*.**

| Gene  Exons | Forward primer (5→3´) | Reverse primer (5→3´) | Fragment size (bp) |
| --- | --- | --- | --- |
| *Hic1* |  |  |  |
| 1-6 | AAAATCGCCCTCTCACAACA | CCCGAGTCCTCTAGGGAAGA | 942 |
| * | ACTCAGCACAAAACCCCAAG |  |  |
| 7 | AAGCGGAGGGGAACAGAC | CAGTACTTGCCATGCCGTTT | 518 |
| 8 | TCAAACGGCATGGCAAGTA | CATAGAGCTCGGCGCAGT | 672**a** |
| 9a | CAGCGGTCAACACCCACT | CGGGTCCTTGTAGCTCTTGT | 932 |
| * |  | TGCTTCATCCAGCGGTAGAG |  |
| * | TACCGACTCCAGACCCGTTT |  |  |
| 9b | TTTGGTGACAACCTGTACGTG | AAACAGTGAGGCCAGGTGAG | 1022 |
| * | GCGCAACCTCATCAGTCATA |  |  |
| * |  | GAAGTCCAGCTTGCCCTTG |  |
| *Myo1c* |  |  |  |
| 1 | AACGTTCCTCCTGGAAAACC | CACCAAGAGACACCCCAGAC | 337 |
| 2-3 | ATGTTCTGTAGCCCGTGTCC | AGATTCCTCACGGTGGTGAC | 583 |
| 4-6 | GTCCACCCTCCTCTCCATCT | GTGCCTGGCATACAATAGGC | 583 |
| 7-8 | CCCAGCTTTACTACAGGGGATA | TCCTCCTCACAGCCCAAATA | 486 |
| 9-10 | CCATCTCCTGCCCTATTTGA | CTCTCTGAGCCACCTCTTGC | 476 |
| 11-12 | GGACAGAAGGGTGTCTGAGG | GGCAGGGGTAAAAGAAGAGC | 440 |
| 13-14 | TGCTTGTGCCTGATTCTGTC | GGGGAAGGGGGTGATAAGTA | 599 |
| 15 | CCCCCTTTTCCTGTACCTGT | CCATGGTGTTGCTACTGTGG | 246 |
| 16-18 | ATGCCCCAGGTCAGAAATC | CTGTCCCCAGTCACACACAC | 491 |
| 19-20 | CCCCAGCCAACCTCCTATAC | CTCCCTCACATGGTCCAGTC | 500 |
| 21-22 | CAGCAAATTCCCCTCCATT | GAGAAAGCCCAAAAGCAGTG | 362 |
| 23-25 | CCCTTTACTCGGGTGTTCCT | CCGACACAGGATCTGAGGAT | 682 |
| 25-26 | TTCTCACCCCAGCTGTGTCT | CTGTACCCCCAAAAGAAGGC | 604 |
| 27-28 | GATGGGCTAGGAAGGGAGTC | TGAGAGGGTGGGGTAGAGTG | 403 |
| 28-30 | CCTTTCTGGCCTTAACCTGA | CTGAGAGGGGAGAACAGAGG | 486 |
| 31-32 | GCTCCCTGACCTCTGTTCTC | CCCATGTCCTTGAGTCACAC | 364 |
| 33-34a | GTGTGTGACTCAAGGACATGG | ATCCCTGTCTGGAAGCTTGA | 537 |
| 33-34b | GCTTTGCTTAGTCCCCTCCT | ATAGTGGGCTGCAAAAGGTG | 441 |
| *Inpp5k* |  |  |  |
| 1 | GCAGTCAGAATCCGGAAGAA | TGATCCCACTGTGGTTTTGA | 279 |
| 2 | GAAACAAACATGCCGACACA | CTCCAAAATGAGGGAGGACA | 305 |
| 3 | TGGTTTTCAGAACCCAGGTC | CATGAAACGCTGAGTGGAGA | 334 |
| 4 | AGGGATTTGCATGTGGTTTC | GTTATGGGGGTGGGTATGC | 352 |
| 5 | AGCTGGGAAGCTGTGTGTCT | CAGATGGGACACCAGGAAAG | 440 |
| 6 | TAAGCTTGTCCTGGCCTTCA | CATTCCGTACCCCAGACATC | 304 |
| 7-10 | AGCTGCTTCCCTGGTCCTAT | TGCCTCTGCCATTTCCTTTA | 521 |
| 11-13 | CTGGGACTGGATCGGACTAT | CAACAGGAATGGGGTCAGTT | 480 |
| 14-15 | TTGTCTTCTGGAAGGAAGGAA | GCTGGAGGAAACTCAGATGC | 548 |

*: intenal PCR-products primer sequencing

**a**: nnnnnn in the reference sequence

Anneling temperature used were 60ºC
