# Supplementary material for: Analysis of an independent tumor suppressor locus telomeric to Tp53 suggested Inpp5k and Myo1c as novel tumor suppressor gene candidates in this region
Source: BMC Genet. 2015 Jul 14;16:80. doi: 10.1186/s12863-015-0238-4 (PMC4501283; doi:10.1186/s12863-015-0238-4)
Supplement: Additional file 3: Table S2. — Primers used for sequencing the genes Hic1, Myo1c and Inpp5k. [file 12863_2015_238_MOESM3_ESM.docx]

**Primers used for bisulphite sequencing of the promotors of *Hic1* and *Myo1C*.** For *Hic1* the predicted CpG island was 877 bp and includes 47 CpG sites. For *Myo1c* the predicted CpG island of 890 bp and includes 55 CpG sites.

| Primer Set | Forward primer (5→3´) | Reverse primer (5→3´) | Fragment size (bp) |
| --- | --- | --- | --- |
| ***Hic1*** |  |  |  |
| 1^st^ set | AGAGTATTTTTGGGGAGTTTTTTTATT | CCAATAAAACACCAAAATCTTAAAC | 328 |
| 2^nd^ set | GAAGGTATAGTTAATAAGAAGTTGTGGG | CCCCATCTAAACAATTTTAAAAAATC | 352 |
| **Myo1c** |  |  |  |
| 1^st^ set* | GTTGGGTAAAATAAAAGGTAGTT | CTAAATAAAAATAAACCTTAAAAC | 617 |
|  |  | ATTAATTCCAAAATAAAACCC | 511 |
| 2^nd^ set* | GGGGTGGGGAGGGAAATTTT | CAACTTTCAAAAACATCTTC | 574 |
|  |  | ACACCCCAAACCCCCAACCTA | 490 |
| 3^rd^ set* | GATTTTTTTAAGTTAAAGGGT | ACCCTAACTATTAACCCTAATTA | 486 |
|  |  | ATCCTCAAATAAAAAAAATCTT | 363 |

* primers used in nested PCR format.
