# Supplementary material for: Analysis of an independent tumor suppressor locus telomeric to Tp53 suggested Inpp5k and Myo1c as novel tumor suppressor gene candidates in this region
Source: BMC Genet. 2015 Jul 14;16:80. doi: 10.1186/s12863-015-0238-4 (PMC4501283; doi:10.1186/s12863-015-0238-4)
Supplement: Additional file 4: Table S3. — Primers used for bisulphite sequencing of the promotors of Hic1 and Myo1C. For Hic1 the predicted CpG island was 877 bp and includes 47 CpG sites. For Myo1c the predicted CpG island of 890 bp and includes 55 CpG sites. [file 12863_2015_238_MOESM4_ESM.docx]

**Primers used for semi quantitative multiplex RT-PCR for Hic1, Inpp5k and Myo1c amplified in a multiplex PCR with β-actin as the internal control.**

| Primer Set | Forward primer (5→3´) | Reverse primer (5→3´) | Fragment size (bp) |
| --- | --- | --- | --- |
| *Hic1* | CAGCGGTCAACACCCACT | TGCTTCATCCAGCGGTAGAG | 376 |
| *Myo1c* | CAGCAGCTCTTCATTGAGC | ATCCAAGATGGAGATGATGC | 150 |
| *Inpp5k* | TATGTCTGGGTCGGTGACAA | GATGGGCACAGTGTGGGT | 152 |
| *β-actin* | CACGGCATTGTCACCAACTG | GATGGGCACAGTGTGGGT | 279 |
