# Supplementary material for: Analysis of an independent tumor suppressor locus telomeric to Tp53 suggested Inpp5k and Myo1c as novel tumor suppressor gene candidates in this region
Source: BMC Genet. 2015 Jul 14;16:80. doi: 10.1186/s12863-015-0238-4 (PMC4501283; doi:10.1186/s12863-015-0238-4)
Supplement: Additional file 5: Table S4. — Primers used for semi quantitative multiplex RT-PCR for Hic1, Inpp5k and Myo1c amplified in a multiplex PCR with β-actin as the internal control. [file 12863_2015_238_MOESM5_ESM.docx]

**SNPs identified in the gene sequencing analysis based on the build RGSC3.4 data.**

| **Gene** | **SNP** | **RNO10 nt Position** | **Annotation** | **Amino acid** | **BDII** | **BN** | **SPRD** |
| --- | --- | --- | --- | --- | --- | --- | --- |
| *Tp53* |  | 56408660 | Intron |  | G | A | G |
| *Hic1* | Deletion | 62472523 | 5´-UTR | - | 6C | 7C | 7C |
|  | SNP1 | 62474413 | Exon | Ala- Thr | A | G | G |
|  | SNP2 | 62475459 | Exon | - | A | C | C |
|  | SNP3 | 62475668 | Exon | - | A | C | C |
|  | SNP4 | 62477031 | 3´-UTR | - | G | G | A |
| *Myo1c* | SNP1 | 62992397 | 5´-UTR | - | C | T | C |
|  | SNP2 | 62992628 | 5´-UTR | - | T | G | T |
|  | SNP3 | 62997009 | Intron | - | G | G | T |
|  | SNP4 | 62999134 | Intron | - | A | G | A |
|  | SNP5 | 63006446 | Intron | - | T | C | C |
